# Liver Dysfunction as a Novel Player in Alzheimer’s Progression: Looking Outside the Brain

**DOI:** 10.3389/fnagi.2019.00174

**Published:** 2019-07-17

**Authors:** Lisbell D. Estrada, Pablo Ahumada, Daniel Cabrera, Juan P. Arab

**Affiliations:** ^1^Bionanotechnology Laboratory, Integrative Center for Applied Biology and Chemistry (CIBQA), Department of Chemical & Biological Sciences, Universidad Bernardo O’Higgins, Santiago, Chile; ^2^Laboratório de Hepatologia Experimental, Gastroenterology Department, Facultad de Medicina, Centro de Envejecimiento y Regeneración (CARE Chile-UC), P. Universidad Catolica de Chile, Santiago, Chile

**Keywords:** amyloid beta, NAFLD, LRP-1, BBB, Alzheimer’s

## Abstract

Alzheimer’s disease (AD) afflicts an estimated 20 million people worldwide and is the fourth-leading cause of death in the developed world. The most common cause of dementia in older individuals, AD is characterized by neuropathologies including synaptic and neuronal degeneration, amyloid plaques, and neurofibrillary tangles (NTFs). Amyloid plaques are primarily composed of amyloid-beta peptide (Aβ), which accumulates in the brains of patients with AD. Further, small aggregates termed Aβ oligomers are implicated in the synaptic loss and neuronal degeneration underlying early cognitive impairments. Whether Aβ accumulates in part because of dysregulated clearance from the brain remains unclear. The flow of substances (e.g., nutrients, drugs, toxins) in and out of the brain is mediated by the blood-brain-barrier (BBB). The BBB exhibits impairment in AD patients and animal models. The effect of BBB impairment on Aβ, and whether BBB function is affected by non-neurological pathologies that impair peripheral clearance requires further investigation. In particular, impaired peripheral clearance is a feature of nonalcoholic fatty liver disease (NAFLD), a spectrum of liver disorders characterized by accumulation of fat in the liver accompanied by varying degrees of inflammation and hepatocyte injury. NAFLD has reached epidemic proportions, with an estimated prevalence between 20% and 30% of the general population. This chronic condition may influence AD pathogenesis. This review article summarizes the current state of the literature linking NAFLD and AD, highlighting the role of the major Aβ efflux and clearance protein, the LRP-1 receptor, which is abundantly expressed in liver, brain, and vasculature.

## Amyloid Beta Role in Alzheimer’s Disease

Alzheimer’s disease (AD) belongs to a large group of neurodegenerative diseases characterized by the pathophysiological brain changes related to the accumulation of misfolded proteins. Specifically, extracellular peptide variants of the amyloid-β (Aβ) accumulate in the form of amyloid plaques or senile plaques, and the intracellular accumulation of neurofibrillary tangles (NTFs) composed by phosphorylated Tau protein (pTau; Bloom, 2014; Héraud et al., 2014; He et al., 2018).

Both are reported to underlie progressive synaptic dysfunction in the AD brain, loss of dendritic spines, and neuronal death (Serrano-Pozo et al., 2011; Busche et al., 2019). Although AD was first described 100 years ago, its precise etiology remains unknown. Efforts to better understand AD have resulted in multiple hypotheses to explain events in AD pathogenesis, for example, the amyloid cascade theory that describes the imbalance between Aβ production and clearance (Selkoe and Hardy, 2016). Here, we provide an overview of the etiology of AD, and the principal concepts that support the critical role of the brain-blood barrier (BBB) and liver in AD development and progression.

In neurons under physiological conditions, Aβ is secreted to maintain normal synaptic function, morphology, and plasticity (Wang et al., 2012; Gouras et al., 2015; Klevanski et al., 2015). Aβ is a by-product generated from cleavage of the amyloid protein precursor (APP). APP plays an important physiological role in regulating γ-aminobutyric acid type B receptor (GABA_B_R) and modulating synaptic transmission and plasticity (Chen et al., 2017; Doshina et al., 2017; Rice et al., 2019). In primary cortical neurons, APP modulates frequency and amplitude of calcium oscillations essential for synaptic transmission (Octave et al., 2013). A mouse model deficient for APP demonstrated that APP is necessary for the synapsis and maintenance of dendritic integrity in the hippocampus (Tyan et al., 2012). Likewise, hippocampal neurons in culture derived from APP knockout mice showed APP is critical for synaptogenesis and dendritic and axonal growth process and regulates substrate adhesion (Southam et al., 2019).

On the other hand, in the amyloidogenic (i.e., disease-causing) pathway, APP is cleaved by β- and γ-secretase to generate Aβ, which accumulates as senile plaques (Hardy and Selkoe, 2002; Konietzko, 2011). AD-related plaques are associated with high levels of soluble oligomeric forms of Aβ (AβOs; Esparza et al., 2013). AβOs comprise soluble dimers and trimers of low molecular weight and soluble oligomeric forms of 12–14 monomers (Mroczko et al., 2018). In addition, these oligomers have been identified as the toxic conformers of Aβ plaques in AD (Jin et al., 2011; Verma et al., 2015). AβOs can diffuse across synaptic membranes (Hong et al., 2014) and trigger a cascade of injurious events in neurons, causing synaptic failure and memory loss (Morris et al., 2014; Brito-Moreira et al., 2017). Moreover, AβOs are associated with dystrophic neurites, reactive astrocytes, and aberrant activation of glutamatergic neurotransmission; the consequence of these changes is neuronal death by excessive neuronal influx of sodium and calcium (Ziegler-Waldkirch and Meyer-Luehmann, 2018). Postsynaptic protein disruption (Lésne et al., 2013) and hippocampal synaptic plasticity impairment by AβOs contributes to memory loss (Müller-Schiffmann et al., 2016). Intracellular AβOs are detectable in cholinergic neurons, suggesting that they play a critical role in cholinergic deficiency (Baker-Nigh et al., 2015). These devastating events not only lead to memory loss and learning impairment in AD patients, but also affect the capacities of reasoning, abstraction, and language (Duyckaerts et al., 2009).

## Blood-Brain Barrier Breakdown and Role of LRP-1 in Alzheimer’s Disease

The blood-brain barrier (BBB) is a specialized structure that supports brain function. This structure supports the brain by regulating electrolyte flux, cerebral blood flow (CBF) and efficient oxygen and metabolite delivery, and restricting entry of potentially toxic and even some therapeutic agents into the brain (Provias and Jeynes, 2014; Andreone et al., 2015; Di Marco et al., 2015). BBB function is mediated by neurovascular units (NVU) comprising neurons, glial cells, pericytes, and brain endothelial cells, which maintain homeostasis of the cerebral microenvironment (Armulik et al., 2011). Brain endothelial cells are an important component mediating the flow between brain and blood by cell-to-cell communications called tight junctions and adherent junctions; these junctions connect cell networks (Deli et al., 2005; Van de Haar et al., 2015) and regulate the paracellular permeability of substances across the BBB (Bowman and Quinn, 2008; Viggars et al., 2011; Kook et al., 2013; Chow and Gu, 2015; Ulrich et al., 2015). Tight junctions proteins ZO-1, Occludin and CLN-5 are key to maintaining BBB integrity (Jiao et al., 2011). ZO-1 joins tight junctions with the actin cytoskeleton, working as accessory proteins (Xiao et al., 2017). Occludin and CLN-5 are transmembrane tight junction proteins involved in signal transduction of cytokines (Haseloff et al., 2015). The high expression of these proteins on brain endothelial cells regulates the transport of essential molecules through the BBB, such as the free and rapid diffusion of oxygen and carbon dioxide (Lin et al., 2015; Pardridge, 2015). Hydrophobic molecules permeate the BBB faster and more easily than hydrophilic molecules, while molecules that are larger than 180 KDa or water-soluble do not penetrate the BBB (Kroll and Neuwelt, 1998; Zlokovic, 2005; Masserini, 2013). For example, the BBB restricts the passage of albumin and immunoglobulins, high-molecular-weight proteins from the peripheral blood circulation (Xiao and Gan, 2013).

Another important component of brain endothelial cells is a complex and specific transport-receptor protein system that also contributes to BBB permeability (Zlokovic, 2011). The luminal side of the BBB contains transporters for specific classes of nutrients, such as glucose and vitamins, and receptors for peptides, proteins, and hormones. These mediators facilitate transport across the BBB from circulating blood into the brain (Deane and Zlokovic, 2007; Simpson et al., 2007). In contrast, the transport system of the abluminal side of the BBB eliminates neurotoxic molecules and metabolic waste (Begley and Brightman, 2003).

Dysfunction of the BBB, therefore, could result in altered permeability. Indeed, age-dependent BBB breakdown at the hippocampus is associated with mild cognitive impairment and correlates with pericytes injury. This finding suggests that the cerebrovascular integrity loss that begins at the hippocampus may contribute to early stages of dementia associated with AD (Montagne et al., 2015). Similarly, early cognitive dysfunction has been associated with capillary damage and BBB breakdown in older adults (Nation et al., 2019).

This breakdown of BBB function may be related to alterations in specific components of the BBB structure. Low-density lipoprotein receptor-related protein 1 (LRP-1) is a membrane receptor that mediates the cellular internalization of multiple ligands. Further, LRP-1 regulates several tight junction proteins in endothelial cells of the BBB (Zhao et al., 2016). Functional LRP-1 is expressed in liver sinusoidal endothelial cells (LECs), highly specialized scavenger cells, and LRP-1 expression contributes to the rapid removal of its blood ligands (Øie et al., 2011). Cell surface LRP-1 and circulating sLRP-1 are needed for brain and systemic clearance of Aβ; however, in AD, both cell surface LRP-1 and circulating sLRP-1 concentrations are dramatically reduced (Sagare et al., 2012). Importantly, these alterations may begin as early as two decades before the manifestation of cognitive impairment symptoms (Beason-Held et al., 2013; Jack et al., 2013; De Strooper, 2014). Clearance of Aβ may also be affected by other pathologies, however.

## Clearance of Aβ at The Periphery: Role of The Liver

Peripheral organs, including the kidney and the liver, play an essential role in the clearance of circulating Aβ. Elimination of Aβ from the circulation may contribute to AD progression, by helping to displace the dynamic equilibrium from Aβ deposited in the senile plaques toward soluble Aβ. This hypothesis is supported by evidence that peritoneal dialysis reduces the circulating levels of Aβ in humans and diminishes AD features in an animal model (Jin et al., 2017). Insufficient clearance of brain Aβ also contributes to the progression of sporadic AD (Wang et al., 2006). As brain Aβ equilibrates with Aβ in plasma, peripheral clearance of Aβ provides a potential approach to facilitate efflux of Aβ from the brain (Liu et al., 2015). Peripheral organs and tissues are key in clearing brain-derived Aβ under physiological conditions (Xiang et al., 2015).

**Figure 1:**
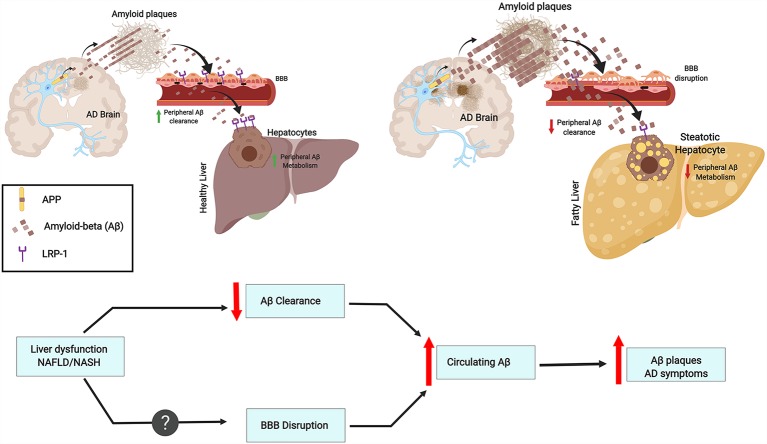
Chronic liver diseases may increase amyloid burden and Alzheimer’s pathology. This contribution results from an imbalance in peripheral amyloid-β (Aβ) clearance as a result of decreased LRP1 levels, general liver dysfunction, and chronic inflammation. These features may worsen blood-brain-barrier (BBB) impairment and contribute to a vicious cycle. As an example, the figure depicts fatty liver disease as a chronic liver condition.

The liver has many functions, one of which is metabolic detoxification. When the liver is under constant injury, as is found in metabolic diseases, it exhibits decreased detoxification capacity. Indeed, the expression of metabolic enzymes decreases in conditions such as obesity, diabetes, and cirrhosis (Rolle et al., 2018). Hepatocytes can act directly on circulating Aβ, promoting its clearance by degradation or through bile excretion. Further, Aβ uptake from circulation can be mediated through LRP-1, which is highly expressed in hepatocytes (Kanekiyo and Bu, 2014). Interestingly, liver dysfunction is accompanied by low LRP-1 hepatic expression and high levels of circulating Aβ. This correlation suggests that Aβ clearance decreases due to low hepatic LRP-1 activity (Wang et al., 2017; see Figure 1).

AD pathophysiology has not been evaluated from a hepatic point of view; yet, the evidence points to a critical role for liver in AD pathogenesis. Aβ levels found in liver samples from AD patients are lower when compared to neurologically healthy controls, raising the possibility that the liver is not properly eliminating circulating Aβ (Roher et al., 2009). This observation is supported by studies where insulin promotes LRP-1 translocation to the cell membrane in hepatocytes, favoring Aβ clearance (Tamaki et al., 2007). The stimulation of LRP-1-mediated liver uptake improves cognitive impairment and decreases Aβ aggregation in the brain in AD transgenic mice (Sehgal et al., 2012).

## NAFLD/NASH Affects Aβ Clearance

Non-alcoholic fatty liver disease (NAFLD) encompasses a spectrum of liver disorders characterized by excessive fat deposition in hepatocytes from individuals who drink little or no alcohol. NAFLD is an umbrella term for several subtypes ranging from isolated hepatic steatosis, or fatty liver, to nonalcoholic steatohepatitis (NASH). NASH is defined by the presence of fatty changes with inflammation and several degrees of hepatocellular injury or fibrosis. Thus, NASH is the aggressive form of NAFLD and can progress to advanced fibrosis and cirrhosis.

NAFLD/NASH is the leading cause of chronic liver disease worldwide and has reached epidemic proportions. Interestingly, most of the deaths in NAFLD patients are not restricted to liver-related morbidity or mortality; rather, cardiovascular disease (CVD) and cancer predominate (Armstrong et al., 2014). Therefore, the presence of fatty liver is not a benign pathology as was historically considered by most clinicians. Indeed, extensive evidence in recent years shows that NAFLD also increases the risk of end-stage liver disease, hepatocellular carcinoma (HCC), liver-related mortality, and all-cause mortality. These observations prompted the idea that NAFLD/NASH, either independently or concomitantly with other metabolic risk factors, determines or even drives extra-hepatic diseases such as CVD, chronic kidney disease, colorectal cancer, endocrine disorders like type 2 diabetes mellitus, osteoporosis, and, indeed, AD. Recent studies have linked insulin-resistance (the key pathophysiological feature of NAFLD) to several of the neurodegenerative mechanisms of AD including oxidative stress, mitochondrial dysfunction, and inflammation, via dysregulated insulin/IGF-1 signaling with attendant impairments in signal transduction and gene expression (de la Monte and Tong, 2014; de la Monte, 2017; Kim et al., 2016).

A network clustering analysis conducted by Karbalaei et al. (2018) indicated that there are 189 genes shared between NAFLD and AD. Further, three main groups of pathways are candidates for contributing to both AD and NAFLD: carbohydrate metabolism, long fatty acid metabolism, and IL-17 signaling pathways (Karbalaei et al., 2018). This suggests that diabetes and obesity might be considered as a risk factor for AD and NAFLD.

One study showed that NAFLD promotes AD in mice (Kim et al., 2016). This study evaluated whether NAFLD induction, through a dietary approach (high-fat diet), promotes the development of AD signs. Brains of HFD-fed mice showed increased levels of neuro-inflammation, characterized by higher levels of cytokines, toll-like receptors, and microgliosis. These features were accompanied by increased plaque formation in a transgenic mouse model of AD. In addition, intense and frequent signs of cerebral amyloid angiopathy (CAA)—a condition characterized by the Aβ deposition in the media and adventitia of small and mid-sized arteries—were observed in mice fed with HFD.

An abnormal lipid metabolism is linked with increased risk for AD development, and the liver plays a crucial role since is the main peripheral organ responsible for lipid metabolism (Fukumoto et al., 2002; Hooijmans and Kiliaan, 2008). Aβ is able to bind Apolipoprotein E (ApoE) and can be cleared from the brain together with cholesterol (Mahley, 1988). Interestingly, ApoE is a ligand of LRP-1 and both are genetically associated with AD and plasma Aβ levels (Kang et al., 2000). This link is intriguing since LRP-1 is suggested to facilitate Aβ clearance from the brain across the BBB (Deane et al., 2004; Sagare et al., 2012; see Figure 1).

## Liver Inflammation and Aβ Levels

Hepatitis B is a liver infection that can become chronic and severe. Interestingly, Hepatitis B Virus (HBV) carriers have significantly higher plasma Aβ levels than non-carriers. Moreover, HBV carrier status is associated with plasma Aβ levels (Jin et al., 2017). Overall infectious burden including cytomegalovirus (CMV), herpes simplex virus type 1 (HSV-1), *Borrelia burgdorferi*, *Chlamydophila pneumoniae* and *Helicobacter pylori* was found to significantly contribute to AD pathogenesis (Bu et al., 2015). However, currently, no epidemiological study has been designed to understand the association between HBV infection and the risk for AD. The effect of chronic inflammation on Aβ clearance is lesser than the effects of HBV infection or liver dysfunction (Liu et al., 2013). Further, although plasma concentrations of cytokines IL-1β and IL-6 are significantly increased in cirrhosis patients and plasma IL-6 levels are correlated with Aβ40 levels (a 40 amino acid proteolytic product of APP cleavage that has gained attention as a biomarker correlating with AD), no association is observed by linear regression between IL-6 and Aβ40 levels. On the other hand, the ratio of AST/ALT, which is an indicator of liver functional impairment (Giannini et al., 1999), is significantly associated with circulating Aβ40 levels (Wang et al., 2017). Furthermore, hepatic dysfunction may lead to a plethora of systemic changes. Approximately 95% of Aβ in the blood is bound to serum albumin (Stanyon and Viles, 2012). The serum albumin pool represents an important reservoir for peripheral clearance of Aβ. Thus, a diminution in blood albumin in cirrhotic patients might contribute to the increase in plasma Aβ levels (see Figure 1).

## Concluding Remarks

AD is a degenerative condition that will afflict an increasing number of people as the global population ages. Unfortunately, current treatments have only transient or modest effects. This article reviews evidence that supports the involvement of liver diseases, a growing health concern, in AD pathogenesis. The liver is the major player in the clearance of Aβ at the periphery, and an impairment of this clearance may shift the delicate Aβ equilibrium toward brain accumulation.

As to the possible role that the liver plays in brain-derived Aβ clearance, the impaired clearance of serum Aβ might contribute to the high Aβ levels in NAFLD patients. This effect is likely due to an intensification of the BBB disruption and drop in LRP-1 levels, the major receptor for Aβ efflux and important effector of clearance.

It is possible that hepatic malfunction contributes to AD in a plethora of non-excluding pathways, including: (i) the failure to maintain Aβ homeostasis at the periphery; (ii) acting as a source of pro-inflammatory cytokines when chronic inflammation follows different types of injury (like virus infection, drug-induced injury, and metabolic diseases); and (iii) through metabolic impairment.

## Author Contributions

LE wrote and edited the manuscript. PA participated in manuscript writing. DC wrote the manuscript and designed figures. JA participated in manuscript writing and editing.

## Conflict of Interest Statement

The authors declare that the research was conducted in the absence of any commercial or financial relationships that could be construed as a potential conflict of interest.
